# Reactivation of immune responses against *Mycobacterium tuberculosis* by boosting with the CpG oligomer in aged mice primarily vaccinated with *Mycobacterium bovis* BCG

**DOI:** 10.1186/1742-4933-10-25

**Published:** 2013-06-22

**Authors:** Keiichi Taniguchi, Takemasa Takii, Saburo Yamamoto, Jun-ichi Maeyama, Sumiko Iho, Mitsuo Maruyama, Narushi Iizuka, Yuriko Ozeki, Sohkichi Matsumoto, Tomohiro Hasegawa, Yuuji Miyatake, Saotomo Itoh, Kikuo Onozaki

**Affiliations:** 1 Department of Molecular Health Sciences, Graduated School of Pharmaceutical Sciences, Nagoya City University, 3-1, Tanabe, Mizuho-ku, Nagoya 467-8603, Japan; 2 Japan BCG Laboratory, 3-1-5, Matsuyama, Kiyose, Tokyo 204-0022, Japan; 3 Department of Safety Research on Blood and Biological Products, National Institute of Infectious Diseases, 4-7-1 Gakuen, Musashimurayama 208-0011, Japan; 4 Laboratory of Host Defense, Faculty of Medical Sciences, Research and Education Program for Life Sciences, University of Fukui, 23-3, Matsuokashimoaizuki, Eiheiji-cho, Yoshida-gun, Fukui 910-1193, Japan; 5 Department of Mechanism of Aging, Institute for Longevity Sciences, National Center for Geriatrics and Gerontology, 35 Gengo, Morioka, Obu, Nagoya, Aichi 474-8522, Japan; 6 Graduated School of Medical Sciences, Nagoya City University, 1, Kawasumi-cho, Mizuho-ku, Nagoya 467-8601, Japan; 7 Department of Food and Nutrition, Sonoda Women’s University, 7-29-1 Minamitsukaguchi-cho, Amagasaki, Kobe, Hyogo, Japan; 8 Department of Bacteriology, Osaka City University Graduate School of Medicine, 1-4-3 Asahimachi, Abeno-ku, Osaka 545-8585, Japan

**Keywords:** *Mycobacterium tuberculosis*, BCG, CpG oligomer, Booster, Aging

## Abstract

**Background:**

*Mycobacterium bovis* bacillus Calmette Guérin (BCG) vaccine, which has been inoculated to more than one billion people world-wide, has significant effect in preventing tuberculous meningitis and miliary tuberculosis (TB) in neonate and early childhood. However, BCG fails to adequately protect against pulmonary TB and reactivation of latent infections in adults. To overcome this problem, adequate booster is urgently desired in adult who received prior BCG vaccination, and appropriate animal models that substitute human cases would be highly valuable for further experimentation.

**Findings:**

The booster effect of the synthesized CpG oligomer (Oligo-B) on aged mice which had been primarily vaccinated with BCG at the age of 4-week old. The specific Th1 type reaction, production of interferon-γ, in response to TB antigens, purified protein derivatives (PPD) and protection against challenge with *Mycobacterium tuberculosis* (MTB) H_37_Rv decreased with increasing age and were not observed in 89-week old mice. In order to rejuvenate the Th1 type response against PPD and protection activity against MTB infection, Oligo-B, which is known to augment Th1 responses, was administered as a booster to 81-90-week old mice (late 50’s in human equivalent) vaccinated with BCG at 4-week old. The boosting with Oligo-B increased the number of CD4^+^ CD44^high^ CD62L^high^, central memory type T cell. Furthermore, the Oligo-B boosting rejuvenated the ability of mice to protect against infection with MTB H_37_Rv.

**Conclusions:**

Th1-adjuvant CpG oligo DNA, such as Oligo-B, may be a promising booster when coupled with BCG priming.

## Introduction

The protective efficacy of BCG vaccine is variable from 0 to 80% in many field trials and uncertain to pulmonary TB in adult [1]. The several reports showed that the effectiveness of prime BCG vaccination would last for around 15–24 years [2,3]. To solve the problem of current BCG vaccine the prime-boost vaccine strategy against TB was investigated strategy [4,5]. In most of the trials in mice, however, intervals between priming and boosting were only 4–8 weeks, which correspond to 2 years in human. Furthermore, the immune response against MTB reaches its peak within several weeks after prime vaccination. Thereby, to evaluate the booster in adult human, it is necessary to investigate the boosting effect in aged animals primed with BCG. The transition of immune system with increasing age has been reported by analyzing the population of T cell subsets [6-8]. However, the reports investigating the duration of efficacy of BCG vaccination and the shift of memory type T cell subsets with increasing age are very few. In this study we investigated the efficacy of prime BCG vaccine with aging by analyzing memory T cell subset, immune responses to TB antigens, and also protection activity against MTB infection in animal model. We also evaluated the effect of boosting with Oligo-B to the protective immunity against TB in BCG-primed aged mice.

## Materials and methods

1. Bacterial strains and cultures

2. Mice and immunization

The methods were described in our previous study [9]. The protocol of animal study was approved by the Ethics Committee of Nagoya City University.

3. Whole blood assay

The whole blood was stimulated with purified protein derivative (PPD) (Japan BCG co., Tokyo, Japan) for 18 h. Then, the supernatant were collected and the amount of interferon gamma (IFN-γ) was measured by enzyme-linked immunosorbent assay (ELISA), using a BD OptEIA^TM^ ELISA set (BD Bioscience, San Jose, CA).

4. Flow cytometric analysis of surface markers

Splenocytes from young and middle-aged mice were washed by FACS buffer and stained with PE rat anti-mouse CD8a (BD Bioscience, San Jose, CA) and FITC rat anti-mouse CD4 (BD Bioscience, San Jose, CA), then analyzed by FACS. MACS^TM^(Miltenyi Biotec, Tokyo, Japan) -purified splenocytes CD4^+^ and CD8^+^ T cells were stained with PE rat anti-mouse CD44 and FITC rat anti-mouse CD62L (BD Bioscience, San Jose, CA), then cells were analyzed by FACS.

5. Protection assay against infection with MTB

The precise method was described in our previous study [9].

6. Splenocytes stimulation

Splenocytes prepared from BCG-immunized with or without Oligo-B (GGGGGGGGGGGG AACGTTGGGGGGGGGGGG) (Nihon Gene Research Laboraroties, Inc., Miyagi, Japan) or Oligo-B negative (GGGGGGGGGGGG ACCGGTGGGGGGGGGGGG) (Nihon Gene Research Laboraroties, Inc., Miyagi, Japan) mice were incubated in a 24-well plate, at a concentration of 5 × 10^5^ cells per well. Cells were stimulated with 10 μg/ml of PPD for 48 h. The productions of IFN-γ in the supernatants of splenocytes were determined by the ELISA set.

7. Statistical analysis

The methods were described in our previous study [9].

## Results

1. The reduction of interferon-γ production from antigen-stimulated T cells of mice immunized with *Mycobacterium bovis* bacillus Calmette Guérin (BCG) with aging

**Figure 1:**
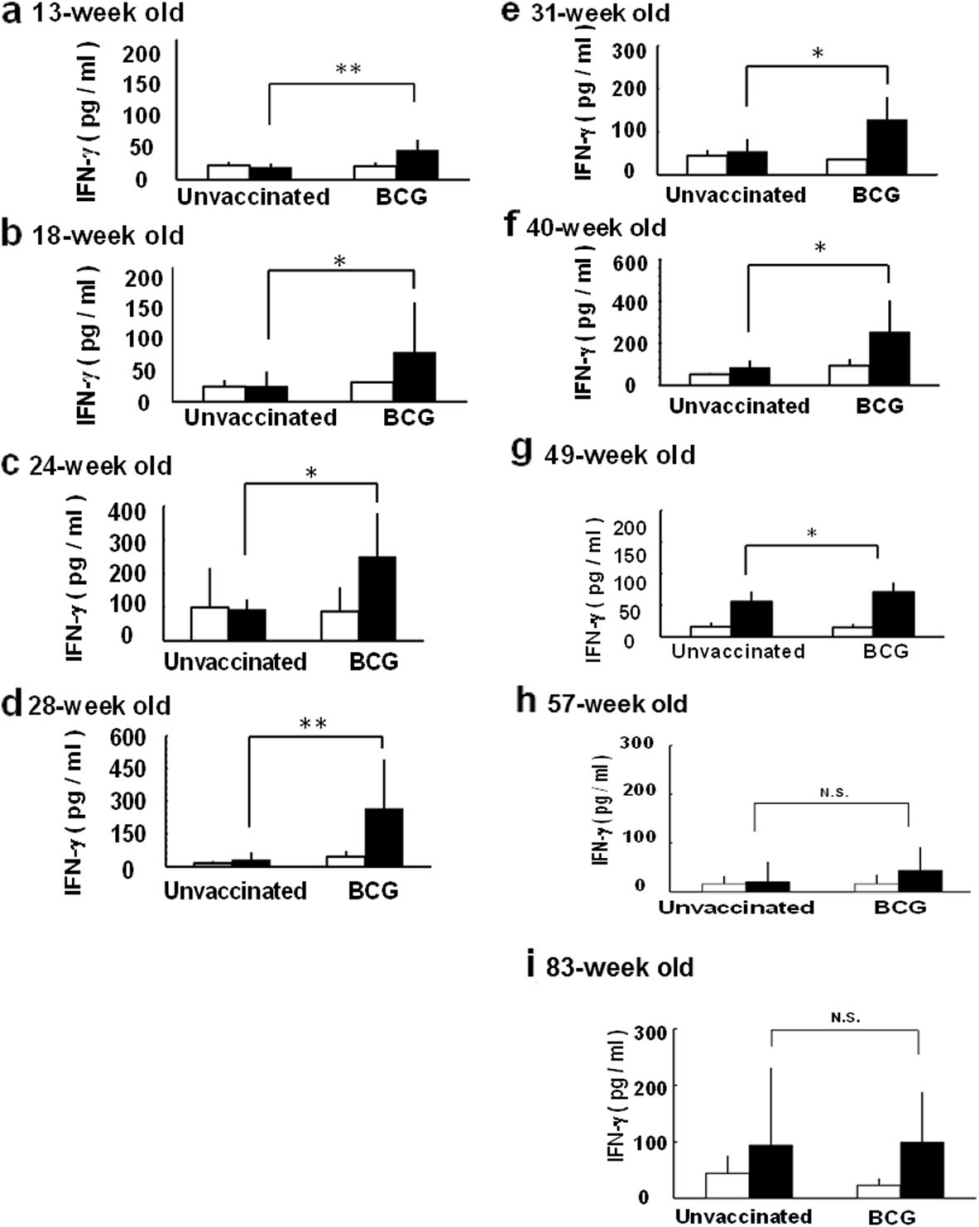
**The change of IFN-γ production from whole blood cells stimulated with PPD in mice with increasing age.** C57BL/6 mice were subcutaneously vaccinated with BCG (10^6^ CFU) or PBS (unvaccinated). Whole-blood obtained from **a**) 13-week old, **b**) 18-week old, **c**) 24-week old, **d**) 28-week old, **e**) 31-week old, **f**) 40-week old, **g**) 49-week old, **h**) 57-week old, **i**) 83-week old was stimulated with purified protein derivative (PPD, solid column) or PBS (open column) and incubated for 24 h. The concentration of interferon-gamma (IFN-γ) from the culture supernatant was measured by ELISA. Results are shown as mean ± SD from groups of 16 animals. ** p < 0.01,* p < 0.05, N.S.: not significant.

**Figure 2:**
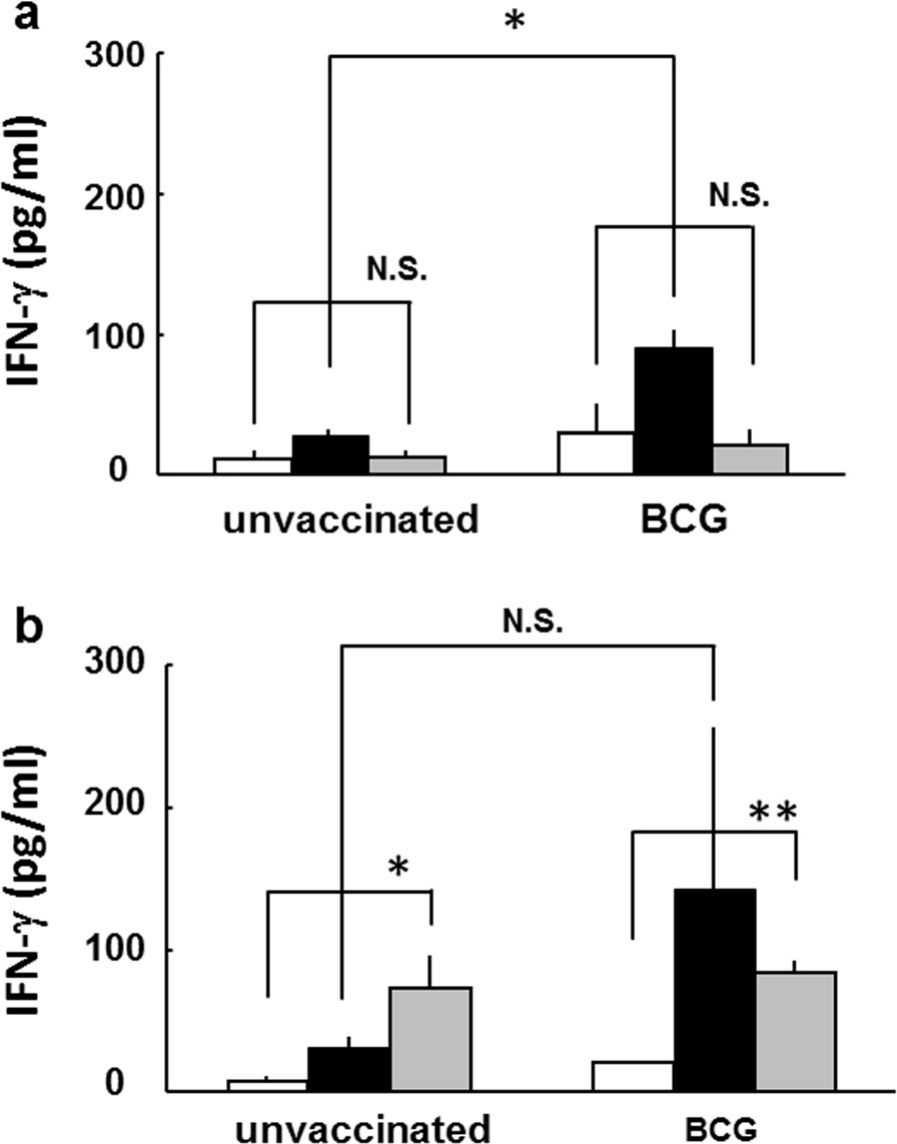
**The IFN-γ production from splenocytes stimulated with OVA in BCG vaccinated young and middle-aged mice.** Splenocytes were prepared from BCG-vaccinated or non-vaccinated **a**)13-week old, **b**) 89-week old mice, and plated in a 24-well plate, at a concentration of 5 × 10^5^ cells per well. Cells were stimulated with 100 μg/ml of ovalbumin (OVA, gray column), 10 μg/ml of PPD (solid column) or PBS (open column) and incubated for 48 h. Then, supernatants were collected and the concentration of IFN-γ was measured by ELISA. Results are shown by mean ± SD from groups of 3 mice. ** p < 0.01, * p <0.05, N.S.: not significant.

The difference of the PPD-induced IFN-γ production between unvaccinated and vaccinated mice with BCG at 4-week old was remarkable until 49-week old mice (Figure 1a-1g), however, it was not significant in 57-week and 83-week old mice (Figure 1h and 1i). The PPD-induced IFN-γ production in unvaccinated mice was comparable to that of BCG vaccinated super aged mice (83-week old) (Figure 1i). The immune response to ovalbumin (OVA), non-specific antigen, increased in both unvaccinated and BCG vaccinated 83-week old mice (Figure 2b). These results suggest that the immune responses specific to tuberculosis antigen decreased, and conversely nonspecific immune responses increased with aging and were supported previous studies about immune senescence with aging [6-8].

2. The change of memory type T cell subsets with aging

We analyzed central type memory T cells (T_CM_), CD44^hi^ CD62L^hi^, and effector type memory T cells (T_EM_), CD44^hi^ CD62L^lo^, in both 30-week and 90-week old mice. CD8^+^ T_EM_ was induced by the immunization with BCG in 30-week mice (Table 1, BCG vaccination; 61.7 ± 0.03 vs un-vaccination; 48.2± 7.95), however, the population of T_CM_ did not change (Table 1, BCG vaccination; 14.7 ± 0.49 vs un-vaccination; 14.0 ± 3.67 ). Marcela et al. reported that the immunization with BCG failed to induce T_CM_[10]. The population of both T_EM_ and T_CM_ in CD8^+^ slightly decreased in BCG-vaccinated 90-week old mice (Table 1, BCG vaccination; 87.51 ± 6.94 vs un-vaccination; 95.90 ± 0.82). These data suggest that the immunization with BCG is not sufficient to induce long term memory type T cells.

3. Boosting with Oligo-B

**Figure 3:**
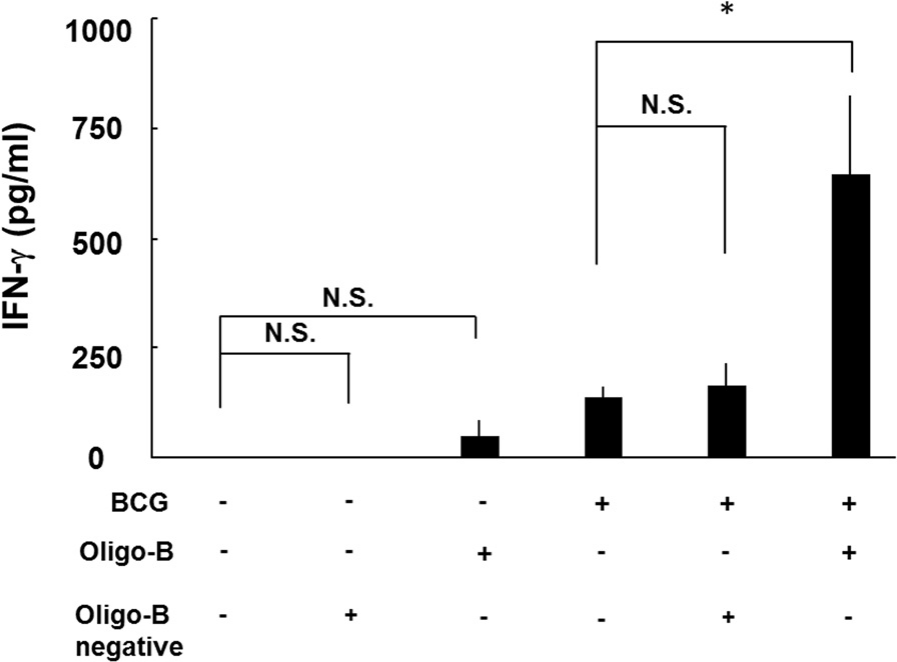
**Oligo-B, but not Oligo-B negative augmented the prime BCG vaccination.** BCG-vaccinated or unvaccinated 30-week aged mice were subcutaneously injected with 50 μg of booster (Oligo-B or Oligo-B-negative), every 2 weeks. Splenocytes prepared from each mouse 2 weeks after 3-times boosting were incubated in a 24-well plate, at a concentration of 5 × 10^5^ cells per well. Cells were stimulated with 10 μg/ml of PPD (solid column) or PBS (open column) and incubated for 48 h. Then, supernatants were collected and the concentration of IFN-γ was measured by ELISA. Results are shown as mean ± SD from groups of three mice. * p < 0.05, N.S.: not significant.

Several researches reported that Th1 type responses, such as production of IFN-γ against PPD, were reduced with aging and the immunization with BCG was not sufficient to induce long term memory T cells [10,11]. We have previously reported that CpG oligomer (Oligo-B) activate Th1 response [12] and enhanced the delayed type hypersensitivity against PPD [13]. Therefore, we investigated the boosting effect of Oligo-B on the reactivation of immune senescence mice. Three times boosting with Oligo-B, but not Oligo-B negative, remarkably augmented the production of IFN-γ from splenocytes stimulated with PPD in the BCG vaccinated mice (Figure 3), and CD4^+^ memory type T cells were strongly induced by the boosting (Table 1, CD4^+^ CD44^hi^ CD62L^high^ in 90-week old mice: after boosting; 7.76±3.26 vs before boosting; 2.85 ± 0.67). These results strongly suggest that the boosting with Oligo-B can effectively reactivate the memory T cells developed by primary vaccination.

4. The effect of boosting with Oligo-B on the protectiveness against MTB in aged mice primarily vaccinated with BCG

**Figure 4:**
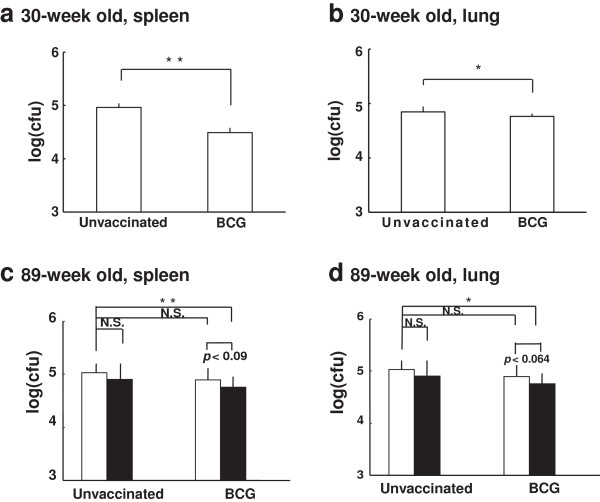
**The protective activity against TB in young and middle-aged mice.** BCG-vaccinated or unvaccinated mice, young (30-week old) (**a**, **b**) or middle-aged (89-week old) (**c**, **d**), were intravenously challenged with 1 × 10^5^ CFU of M. tuberculosis strain H_37_Rv. Middle-aged mice (**c**, **d**) were non-boosted (open column), 3-times boosted by Oligo-B (solid column). Four weeks after challenge, the bacterial numbers in the spleen (**a**, **c**) and lung (**b**, **d**) were determined by colony assay. Data represent mean ± SD from 8-10 mice. The results of Oligo-B-boosted mice were obtained from 4 mice. ** p < 0.01, * p < 0.05, N.S.: not significant.

The bacterial numbers of MTB H_37_Rv challenged intravenously were reduced in the spleen and lung by BCG vaccination in 30-week old mice (Figure 4a and 4b). However, at 89-week old, the reduction of challenged MTB number was not significant as compared to unvaccinated control mice (Figure 4c and 4d, open column). After three times boosting of Oligo-B on the 84-week old mice vaccinated with or without the prime BCG vaccination, these mice were challenged with MTB H_37_Rv intravenously at 90 weeks old. The bacterial numbers decreased in the lungs and spleens from the mice vaccinated prime BCG plus three times boosting with Oligo-B (Figure 4c and 4d, unvaccinated (open column) vs BCG plus OligoB (solid column)). These data indicate that Oligo-B rejuvenates the weakened protective immunity against MTB infection in BCG-primed aged mice.

**Table 1:** **Transition of memory T cells in BCG immunized mice with aging and retrieval effect of the boosting with Oligo-B on memory T cells**^**$**^

			**CD4**^**+**^		**CD8**^**+**^	
**Age**		**Immunization**	CD44hi CD62L^**^		CD44hi CD62L**	
**in mice**	in human equivarent^*^	**with BCG**^**&**^	**low (T**_**EM**_**)**	**high (T**_**CM**_**)**	**low (T**_**EM**_**)**	**high (T**_**CM**_**)**
30-week	late 10's	+	72.2 ± 0.82	15.6 ± 0.80	61.7 ± 0.03	14.7 ± 0.49
		-	74.5 ± 1.23	13.8 ± 0.35	48.2 ± 7.95	14.0 ± 3.67
81-90-week	late 50's	+	85.75 ± 5.91	2.85 ± 0.67	87.51 ± 6.94	2.07 ± 0.12
		-	93.03 ± 0.56	5.95 ± 0.68	95.90 ± 0.82	3.38 ± 1.94
**Boosting with Oligo-B**						
30-week	late 10's	+	73.6 ± 1.92	17.3 ± 0.32	53.7 ± 10.3	9.95 ± 0.15
		-	78.1 ± 3.89	14.6 ± 0.88	51.8 ± 0.48	10.2 ± 0.11
81-90-week	late 50's	+	89.12 ± 0.62	7.76 ± 3.26	95.29 ± 0.82	2.66 ± 1.08
		-	85.82 ± 0.01	3.82 ± 0.42	92.07 ± 1.41	3.98 ± 0.12

^$^Splenocytes were prepared from the aged mice indicated in the table and stained with specific antibodies to memory T cells (CD44, CD62L), and then analyzed by a flow cytometer. Precise procedures were described in the Section of Materials and methods.

^*^The age in human equivalent was applied to survival curve of mice.

^&^Mice were administered transdermally with BCG at 4-week old.

^**^Effector memory T cell (T_EM_), CD44^hi^ CD62L^lo^; central memory T cell (T_CM_), CD44^hi^ CD62L^hi^.

N.D.: not determined.

## Discussion

In this study, we first analyzed the age related changes of immune responses to MTB antigens, in super aged mice (up to 89-week old) vaccinated with BCG in 4-week old. The IFN-γ production from whole blood cells from C57BL/6 mice immunized with BCG at 4-week old increased up to 13-week old and sustained to 49-week old (Figure 1). These results are consistent with our previous report [14]. We also found the accumulation of CD8^+^ T cells (data not shown) and increased IFN-γ production from splenocytes stimulated with OVA with increasing age (Figure 2). Kim et al. reported that the non-specific type immune responses increased with increasing age [15]. The productions of tumor necrosis factor-α and IL-6 both in healthy individuals and patients suffering from age related diseases increased with aging [16]. These studies indicate that the age related immunological changes and immune senescence in human are commonly observed in mice, and duration of initial BCG vaccine would be extinguished up to 57-week old.

Next, we investigated the reactivation of the protective immunity against MTB infection in the immune senescent mice by Oligo-B boosting (Figure 3). The CpG oligomer was used as adjuvant conjugated with BCG [17] or MTB antigens, such as Ag85B [18], MPT-51 [19], and MTB culture filtrate proteins [20]. The synthesized CpG oligomer, Oligo-B, induced antigen presentation through toll-like receptor 9 (TLR-9) signaling in plasmacytoid dendritic cells [21] and the production of Th17 cytokines [22], which is known to play an important role in host defense against MTB infection [23]. Therefore, these studies indicated that CpG oligomer is a good inducer of IFN-γ and IL-17 and reactivates acquired immunity. In fact, the production of IFN-γ was reactivated by three times boosting of Oligo-B in the super aged mice (Figure 3), therefore, Oligo-B could generate the number of the memory T cell by IFN-γ and IL-17.

Several studies reported that CD4^+^ memory T cells were induced by BCG vaccination [24,25]. In our study the number of T_CM_, CD44^hi^, CD62L^hi^, in CD4^+^ was highly induced by the boosting with Oligo-B in the immune senescent 81-90-old mice (Table 1, 2.85 ± 0.67 vs. 7.76 ± 3.26) and protection activity against MTB was rejuvenated (Figure 4). These results suggest that Oligo-B could induce T_CM_ in CD4^+^ which improved protection activity against MTB infection.

In conclusion, this is the first report indicating that Oligo-B boosting can rejuvenates the number of T_CM_ in CD4+ and reactivate the protection immunity against MTB infection in the immune senescent state mice formerly vaccinated with BCG. This report also provides basic information to explore the prime-booster strategy for preventing TB in adult.

## Abbreviations

TB: Tuberculosis; MTB: *Mycobacterium tuberculosis*; BCG: *Mycobacterium bovis* bacillus Calmette Guérin; IFN: Interferon; IL: Interleukin; CFU: Colony forming unit; PPD: Purified protein derivatives.

## Competing interests

The authors declare that they have no competing interests.

## Authors’ contributions

TT, JM, SY SI, YO, SM and KO designed and planned the research. KT, TT, MM, TH, YM, and SI performed the collection of serum and cytokine analysis. TT, KT, TH, YM and NI performed infectious experiments and counting colonies of bacilli form infected organs. KT and YM performed FACS analysis. JM and SY supplied the BCG vaccine. JM, SI and SY supplied Oligo B. All authors read and approved the final manuscript.
